# Predictors of Urinary 3-Phenoxybenzoic Acid Levels in 50 North Carolina Adults

**DOI:** 10.3390/ijerph13111172

**Published:** 2016-11-23

**Authors:** Marsha Morgan, Paul Jones, Jon Sobus, Dana Boyd Barr

**Affiliations:** 1 United States Environmental Protection Agency, 109 TW Alexander Drive, Research Triangle Park, NC 27711, USA; paul-a.jones@epa.gov (P.J.); sobus.jon@epa.gov (J.S.); 2 Department of Environmental Health, Rollins School of Public Health, Emory University, Atlanta, GA 30322, USA; dbbarr@Emory.edu

**Keywords:** adults, insecticides, exposure, determinants, urine, biomarkers

## Abstract

Limited data are available on the non-chemical stressors that impact adult exposures to pyrethroid insecticides based on urinary biomonitoring. The urinary metabolite, 3-phenoxybenzoic acid (3-PBA), is commonly used to assess human exposure to a number of pyrethroids. In a further analysis of published study data, we quantified urinary 3-PBA levels of 50 adults over a single, 24-h sampling period and examined the associations between the biomarker measurements and selected non-chemical stressors (demographic, lifestyle, and dietary factors). A convenience sample of 50 adults was recruited in North Carolina in 2009–2011. Participants collected individual urine voids (up to 11) and filled out activity, food, and pesticide use diaries over a 24-h sampling period. Urine voids (*n* = 326) were analyzed for 3-PBA concentrations using high-performance liquid chromatography-tandem mass spectrometry. 3-PBA was detected in 98% of the 24-h composited urine samples. The geometric mean urinary 3-PBA level was 1.68 ng/mL in adults. Time spent outside (*p* = 0.0006) was a highly significant predictor of natural log-transformed (ln) urinary 3-PBA levels, while consumption of coffee (*p* = 0.007) and breads (*p* = 0.019) and ln creatinine levels (*p* = 0.037) were significant predictors of urinary 3-PBA levels. In conclusion, we identified specific factors that substantially increased adult exposures to pyrethroids in their everyday environments.

## 1. Introduction

Pyrethroids are a class of synthetic insecticides that were developed over 40 years ago [1,2]. Over the past decade, the popularity and the use of pyrethroids in the United States (U.S.) to control insects at homes and on agricultural fields have dramatically increased [3,4,5]. More than 20 pyrethroids are currently registered for use in residential and/or agricultural settings by the U.S. Environmental Protection Agency (EPA) [6]. At the moment, no data exist in the literature on the total amount of pyrethroids that are being applied in these settings annually [6]. Several studies have found a number of these pyrethroids in samples of dust, surface wipes, food, and/or beverages at U.S. homes [7,8,9,10,11]. This information suggests that adults are likely being exposed to pyrethroid insecticides through the dietary, non-dietary, and dermal routes. However, research has indicated that dietary ingestion is likely the dominant exposure route of adults to pyrethroids in residential environments [12,13].

In humans, the lipophilic pyrethroids are rapidly absorbed and metabolized into more polar products and are primarily excreted in urine with elimination half-lives <12 h [14,15]. 3-Phenoxybenzoic acid (3-PBA) is a major urinary biomarker for seven of the currently used pyrethroids (i.e., cyhalothrin, cypermethrin, deltamethrin, esfenvalerate, fenpropathrin, permethrin, and tralomethrin) that can be applied in residential and/or agricultural settings [8,16]. Several published studies have found measurable levels of 3-PBA in the urine of non-occupationally exposed adults across the U.S. [11,13,17,18,19,20]. In these studies, 3-PBA was frequently detected in adult urine samples with median levels ranging from 0.30 ng/mL to 18.3 ng/mL.

Few U.S. studies have examined the impact of non-chemical stressors (i.e., demographic, lifestyle, or dietary factors) on the non-occupational exposure of adults to pyrethroids using urinary biomonitoring [12,17,18,19]. Examples of demographic factors are age, sex, and educational level, and examples of lifestyle factors are pet ownership, pesticide use, and time spent outdoors. Dietary factors include consumed foods and beverages like vegetables, fruits, meats, dairy, grains, milk, and juices. A study conducted by Berkowitz et al. [17] showed that education level, marital status, residential ownership, and sampling season were strong predictors (*p* < 0.05) of urinary levels of 3-PBA in 386 New York women in 1998–2001. Reiderer et al. [12] also reported that the consumption of certain food items (wine, orange juice, biscuits, rice, bacon, chicken patties, spinach, salsa, lettuce, broccoli, salty snacks, or peanut butter) were significantly associated (*p* < 0.05) with urinary 3-PBA concentrations in 1087 adults in the 1999–2002 U.S. National Health and Nutritional Examination Survey (NHANES). In addition, McKelvey et al. [18] showed that race, sex, and the weekly consumption of green vegetables (e.g., lettuce, green peppers, and cucumbers) were significant predictors (*p* < 0.05) of urinary 3-PBA concentrations in 1452 New York adults in 2004. In a more recent study conducted by Morgan [19], sampling season, pet ownership (dog or cat), and removal of shoes before entering the home were strong predictors (*p* < 0.05) of urinary 3-PBA levels in 121 Ohio adults in 2001. These above studies provide evidence that certain non-chemical stressors can substantially influence the exposures of some U.S. adults to pyrethroids. As data are limited, more research is necessary to identify the important non-chemical stressors that markedly impact adult exposure to pyrethroid insecticides.

In previous work from the “Pilot Study to Estimate Human Exposures to Pyrethroids using an Exposure Reconstruction Approach” (Ex-R study), we quantified the distributions and variability of several pyrethroid metabolites levels including 3-PBA, *cis*-3-(2,2-dichlorovinyl)-2,2-dimethylcyclopropane carboxylic acid (*cis*-DCCA), *trans*-3-(2,2-dichlorovinyl)-2,2-dimethylcyclopropane carboxylic acid (*trans*-DCCA), and 2-methyl-3-phenylbenzoic acid (MPA) in several spot (bedtime and first-morning void, FMV) and 24-h urine samples in 50 adults at home over a six-week monitoring period in North Carolina (NC) [20]. Only 3-PBA was frequently detected (>50%) in the adults’ urine samples. At the moment, it remains unclear whether certain non-chemical stressors substantially influence the residential exposures of Ex-R adults to pyrethroids (that breakdown to form 3-PBA in urine). Therefore, for this current work, the objectives were to quantify the distribution of 3-PBA levels in 50 Ex-R adults over a single, 24-h sampling period and to examine associations between selected demographic, lifestyle, and dietary factors and urinary 3-PBA concentrations.

## 2. Materials and Methods

### 2.1. Study Participants

The Ex-R study investigated the cumulative exposures of 50 adults to several current-use pyrethroids in residential settings. The study design and sampling methodology were described in-depth in Morgan et al. [20]. Briefly, this study was conducted at the U.S. EPA’s Human Studies Facility (HSF) in Chapel Hill, NC and at the participants’ residences within a 40-mile radius of the HSF. Adults aged 19 and 50 years old collected their own urine samples (spot and 24-h) and completed daily pesticide use, food, and activity diaries during weeks 1, 2, and 6 over a six-week monitoring period in 2009–2011. The study protocols and procedures were approved by the University of North Carolina’s Institutional Review Board (project number 09-0741) in 2009. Written informed consent was obtained from the adults prior to study participation. The adults were asked to perform their normal daily activities while participating in this study. None of the participants reported working in occupations (e.g., farmers, pest control operators etc.) that could expose them to pesticides.

For this current work, we used the urine data records of the 50 Ex-R adults that occurred over a 24-h period on days 1–2 of sampling week 1. For each participant, the 24-h urine sample was collected starting after the FMV on day 1 and ending after the FMV on day 2. Up to 11 individual urine voids were collected by each participant during the 24-h sampling period. We selected this 24-h time period as it had the highest completion rates (88%) for urine sample collection by the participants [20]. In addition, we used the participants’ available demographic data records and corresponding diary data records (pesticide use, 24-h activity, and 24-h dietary) for this analysis. The completion rates for the three types of diaries were similar (>95%) across all sampling days.

### 2.2. Diary Collection

The pesticide use diary was completed by the participants at the end of sampling week 1. This diary was used to record the application(s) of any insecticides that occurred at the participants’ homes within the month prior to field sampling and during sampling week 1. The activity and food diaries were completed by the participants over a 24-h period during sampling day 1 of week 1. The 24-h food diary was used to record the consumption of all foods and beverages by the participants over three consecutive time frames (04:00 a.m.–11:00 a.m.; 11:00 a.m.–05:00 p.m.; and 05:00 p.m.–04:00 a.m.). The 24-h activity diary was used to record the amount of time (in 30-min intervals) the participants spent outside the home, inside the home, or away at other locations (e.g., work). These three diaries provided specific types of participant data, including lifestyle factors (i.e., time spent outdoors and insecticide use) and dietary factors (consumption of individual foods and beverages).

### 2.3. 24-h Urine Sample Collection

The participants collected urine voids in separate 1 L polypropylene containers (Government Scientific Source, Reston, VA, USA). The entire urine void was collected for each urine sample. The individual urine voids were stored in portable thermoelectric coolers (Vinotemp International, Irvine, CA or Princess International, Restin, VA, USA). The participants returned the coolers containing the urine samples to the HSF in the morning (08:00 a.m.–11:00 a.m.) of day 3 of week 1. A total of 326 urine samples were collected by the participants during the 24-h sampling period. For each urine void, multiple 8 mL aliquots of urine were transferred into separate 10 mL polypropylene cryovials (Simport, Saint-Mathieu-de-Beloeil, QC, Canada) and stored in freezers at −80 °C until analysis.

### 2.4. Analysis of the 24-h Urine Samples

The chemical analysis of the urine aliquots, including quality assurance and quality control procedures, was described in detail in Morgan et al. [20]. Briefly, 0.5 mL of urine was removed from each sample aliquot and transferred to individual vials. The aliquots (*n* = 326) were hydrolyzed with β-glucuronidase/sulfatase (Sigma Aldrich, St. Louis, MO, USA) for about 16 h and then subjected to solid phase extraction using a 3 cc 60 mg OASIS HLB cartridge (Waters Corporation, Milford, MA, USA). Finally, the extracts were evaporated with a TurboVap LV Evaporator (Biotage, Charlotte, NC, USA) and 100 µL of a solution (30% methanol/70% deionized water) was added to each vial. The extracts were analyzed for levels of 3-PBA using a high-performance liquid chromatography-tandem mass spectrometry (Agilent Tech., Waldbonn, Germany). The estimated limit of quantitation (LOQ) for 3-PBA was 0.25 ng/mL in urine. In addition, individual urine aliquots were analyzed for levels of creatinine using a modified Jaffe method [20,21].

### 2.5. Statistical Analysis of the Data

Summary statistics (GraphPad Prism version 5.04; GraphPad Software, San Diego, CA, USA) including detection frequency, arithmetic mean/standard deviation, geometric mean, percentiles (25th, 50th, 75th, and 95th) and range were computed for concentrations of 3-PBA in the participants’ urine over the 24-h sampling period as unadjusted (ng/mL) and creatinine-adjusted (ng/mg) values. All urine data values below the LOQ were replaced with the formula LOQ$/\sqrt{2}$ [22]. For each participant, their average 24-h urinary 3-PBA concentration and average creatinine concentration values were used for the summary statistics.

Creatinine-adjusted urine values were calculated using the Equation (1) [23]:

Creatinine-adjusted urine value (ng/mg) = 100 mL/dL × 3-PBA level (ng/mL)/creatinine level (mg/dL)
(1)

Creatinine-adjusted urine values were previously found to introduce additional measurement variability for adults (both within and between) [20]. Thus, we only used unadjusted urinary 3-PBA levels for the subsequent statistical analyses. The distribution of the Ex-R adults’ unadjusted urinary 3-PBA levels were found to follow a non-normal distribution according to the Shapiro–Wilk normality test. Therefore, the adults’ urinary 3-PBA levels were natural log-transformed (ln) in order to normalize this distribution. Since the 3-PBA urine data were log-normally distributed, a two-sample *t*-test or an analysis of variance (ANOVA) were used to examine the bivariate associations between selected demographic, lifestyle, and dietary factors and ln urinary 3-PBA concentrations. For this analysis, demographic factors were sex, age, race, and body mass index (BMI). Lifestyle factors included sampling season, insecticide use at home (within one month of field sampling), and time spent outside at home. Dietary factors were specific foods and beverages that were frequently consumed by the participants (10 or more people) over the 24-h sampling period. The food items included were apples, bananas, strawberries, other berries, beans, carrots, leafy vegetables, onions, potatoes, peppers, tomatoes, baked or grilled chicken/turkey, fried chicken or chicken nuggets, ham or lunch meats, cheeses, yogurts, breads, cereals, spaghetti/other pastas, salty snacks, sweet snacks, and nuts/nut butters. The beverages consisted of soft drinks (sugar or sugarless), coffee, fruit juices, milk, and water.

Using SAS version 9.4 (SAS Institute Inc., Cary, NC, USA), a multiple regression model was constructed to collectively examine the relationships between the participants’ ln urinary 3-PBA concentrations (dependent variable) and selected demographic, lifestyle, and dietary factors (independent variables). In this full model, we included all factors that had individual *p*-values < 0.2 in our above bivariate analyses. We also included, in this full model, ln-transformed creatinine concentration as an additional independent variable in order to adjust for variable dilutions in the urine samples [24]. In our multiple regression analysis, we performed a sequential, backwards elimination procedure using PROC GLM in SAS. We retained all variables in our final regression model that changed the *R*^2^ value by ≥20% or had a *p*-value of <0.05.

## 3. Results

### 3.1. Urinary 3-PBA Concentrations

Table 1 provides the summary statistics for the unadjusted (ng/mL) and adjusted (ng/mg-creatinine) concentrations of 3-PBA in the urine of the 50 Ex-R adults over the 24-h sampling period. The results showed that 3-PBA was frequently detected (98%) in the adults’ 24-h composited urine samples. The participants’ geometric mean (GM) 3-PBA levels were 1.68 ng/mL for unadjusted measurements and 1.74 ng/mg-creatinine for adjusted measurements. In comparison to our previous work [20], the Ex-R participants’ GM 3-PBA concentrations were lower for both unadjusted measurements (0.98 ng/mL) and adjusted measurements (1.30 ng/mg-creatinine) over a six-week monitoring period.

### 3.2. Predictors of Urinary 3-PBA Concentrations

**Figure 1:**
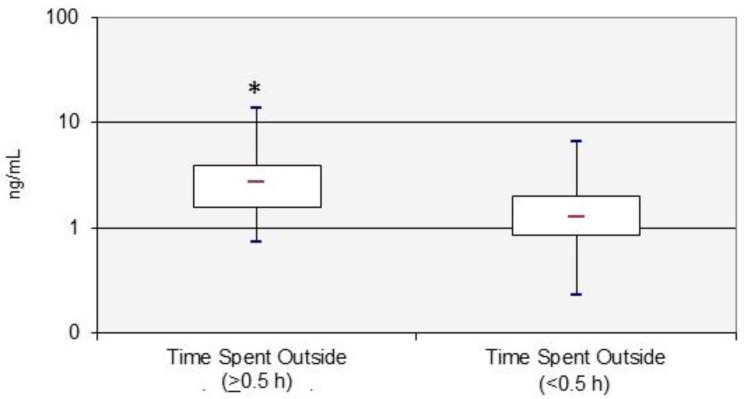
Box-and-whisker plot of the urinary 3-phenoxybenzoic acid (3-PBA) levels of adults that spend ≥0.5 h versus to <0.5 h outside at home over the 24-h sampling period. * *p* = 0.002.

The bivariate associations between the selected demographic, lifestyle, and dietary factors and urinary 3-PBA concentrations are presented in Table 2 and Table 3. Across all of these factors, we found that only one lifestyle factor—time spent outside at home—significantly (*p* < 0.05) influenced the participants’ urinary 3-PBA levels. Urinary 3-PBA concentrations were significantly higher (*p* = 0.002) in adults that spent ≥0.5 h (GM = 2.80 ng/mL) compared to <0.5 h (GM = 1.32 ng/mL) outside at home (Figure 1).

Using the Pearson correlation test, we investigated correlations between all independent variables included in the final models, and all correlation coefficients were below 0.4 (or above −0.4). Variables that were significantly correlated with each other were coffee and beans (*R* = 0.31, *p* = 0.030), fruit juices and beans (*R* = −0.29, *p* = 0.039), and fruit juices and soft drinks (sugar) (*R* = 0.36, *p* = 0.011).

In Table 4, our final regression model showed that time spent outside (*p* = 0.0006) was a highly significant predictor of urinary 3-PBA levels and coffee consumption (*p* = 0.007), bread consumption (*p* = 0.019), and creatinine levels (*p* = 0.037) were significant predictors of urinary 3-PBA levels. In addition, our model showed that fruit juice (*p* = 0.066) and bean (*p* = 0.100) consumption were marginally significant predictors of the adults’ urinary 3-PBA levels. Of these six factors, only the consumption of breads (i.e., rolls, English muffins, biscuits, bagels, and scones) was negatively correlated with urinary 3-PBA levels. Overall, these above six factors collectively explained 44% of the variability of 3-PBA concentrations in the Ex-R participants’ urine samples.

## 4. Discussion

In the U.S., limited data are available on the non-occupational exposures of adults to pyrethroid insecticides based on urinary biomonitoring [12,13,17,18,19]. In our current study based on the 24-h composited urinary 3-PBA data, we found that 98% of the Ex-R adults were exposed to one or more pyrethroids in NC. The Ex-R participants’ GM urinary 3-PBA level was 1.68 ng/mL. In comparison to our study, the 2009–2010 U.S. NHANES, a population-based study, reported a much lower GM 3-PBA level of 0.42 ng/mL in the (spot) urine samples of 1296 adults aged 20–59 years [25]. Other studies conducted in the Caribbean, China, Japan, Poland, and Puerto Rico have also reported GM 3-PBA concentrations ranging between 0.20 and 1.77 ng/mL in adult (spot) urine samples [26,27,28,29,30]. These data confirm widespread exposure of adults to pyrethroid insecticides worldwide.

Previous biomonitoring studies have mostly collected one spot urine sample from adult participants. Recent research has raised concerns on whether a single urinary 3-PBA measurement accurately reflects a person’s exposure to pyrethroids over a day or longer [20,31,32]. The major assumption in these studies has been that the measured 3-PBA concentration in an individual’s spot urine sample is fairly equivalent to their average 24-h concentration [33]. However, due to the short elimination half-lives (<12 h) of pyrethroids, spot urinary 3-PBA measurements likely only reflect a person’s recent exposure, and they can have considerable intra-individual variability over a day [15,20,24]. Currently, few published studies have quantified the levels of 3-PBA in both spot and 24-h urine samples of adults from the general population [20,32]. Wielgomas [32] reported median urinary 3-PBA levels of 0.26, 0.32, and 0.27 ng/mL in spot, FMV, and 24-h urine samples, respectively, in seven Polish adults over a week in 2011. In that study, the intra-class correlation coefficient (ICC) estimates showed good reproducibility (0.55–0.68) of 3-PBA in spot, FMV, and 24-h urine samples. In contrast, we showed in our previous work [20] that median urinary levels of 3-PBA were higher at 0.82 ng/mL, 0.77 ng/mL, and 0.92 ng/mL in bedtime, FMV, and 24-h samples in 50 Ex-R adults over a six-week monitoring period in NC in 2009–2011. Our ICC estimates for 3-PBA showed poor reproducibility for all unadjusted (<0.07) and creatinine-adjusted (<0.22) urine measurements over a day, week, or six weeks. In our current work, Ex-R adults’ median 3-PBA levels were higher at 1.41 ng/mL for a single, 24-h pooled urine sample. This information questions the validity of using a spot urinary 3-PBA measurement to quantitatively characterize the daily exposure and potential health risks of adults to pyrethroids. As data are limited, more research is needed to quantify the levels of 3-PBA in both spot and 24-h urine samples of adults.

A limited number of published studies have examined the influence of demographic, lifestyle, and/or dietary factors on spot urinary 3-PBA measurements in adults, and none using 24-h measurements [12,17,18,20,28,34,35]. The regression modeling results in this current study showed that time spent outside, consumption of coffee, beans, fruit juices, and breads, and creatinine levels together explained 44% of the variability of 3-PBA concentrations in the participants’ urine samples. An important study result was that urinary 3-PBA concentrations were significantly greater (*p* = 0.0006) in Ex-R adults that spent ≥0.5 h compared to <0.5 h outside at home (Figure 1). Based on the pesticide use data records, only four participants reported homeowner applications of pyrethroids (that could form 3-PBA in urine) occurring outside their homes within one month of field sampling and none of them during sampling week 1. This information suggests that some of the Ex-R participants were likely being exposed (i.e., dermal and/or nondietary routes) to unknown sources of pyrethroid residues, perhaps on lawns or common areas, while outside their residences. Morgan [19] also reported finding that urinary pesticide biomarker (2,4-D) levels were significantly higher (*p* = 0.038) in Ohio adults who spent ≥3 h versus <3 h outside their residences in 2001. Another important result was that urinary 3-PBA concentrations were significantly greater (*p* = 0.007) in participants that drank coffee (all varieties) compared to those that did not. These data suggest that this popular beverage may be a significant source of dietary exposures of adults to some pyrethroid(s). Our results are supported by Preedy [36] who reported pyrethroids as one of the major classes of insecticides that are applied on coffee crops, globally. Only one other published study was found by Fortes et al. [35] that reported arithmetic mean urinary 3-PBA levels that were not significantly (*p* = 0.78) higher in Italian adults that consumed coffee >1 time a day (*n* = 39) compared to ≤1 time a day (*n* = 15). The differences between the two studies’ results may possibly be attributed to the higher percentage of Italian adults (>72%) compared to Ex-R adults (46%) that consumed coffee. Therefore, more research is necessary to determine if adults are exposed to significant levels of pyrethroid insecticides in coffee beans or in brewed coffees. Lastly, another interesting result was that urinary 3-PBA concentrations were significantly higher (*p* = 0.019) in Ex-R participants that did not consume breads compared to those that consumed breads (i.e., rolls, English muffins, biscuits, bagels, scones, and white/wheat). Correlations between bread intake and consumption of other food items were investigated as a means of explaining the observed negative effect. We found that only nuts/nut butters were somewhat correlated with breads (*r* = 0.353, *p* = 0.012). It is possible that another food item(s) (not captured in the food diary/statistical analysis) could be inversely related to bread consumption, and positively associated with urinary 3-PBA levels.

Previous studies have shown that several pyrethroids and 3-PBA were concurrently detected in residential media and in (solid) foods [8,11,20,37,38]. This research raises concerns that human exposure to 3-PBA, particularly through dietary ingestion, could be substantially contributing to the total urinary 3-PBA levels measured in adults in previous studies. For the Ex-R adults, 49% of their 782 duplicate-diet solid food samples contained one or more pyrethroids over the six-week monitoring period [39]. 3-PBA was detected in less than 1% of these food samples. Beverage samples, however, were not collected in this study because of participant burden and budget constraints. Therefore, it remains unclear whether the participants’ consumed beverages samples (i.e., coffee and fruit juices) contained measureable levels of these pyrethroids and/or 3-PBA. Future research is needed to determine the concurrent levels of pyrethroids and their environmental degradates (used as urinary biomarkers) in consumed beverages.

## 5. Conclusions

In this study, the urinary 3-PBA data showed that most of the Ex-R participants (98%) were exposed to one or more pyrethroids at home over the 24-h sampling period in NC. Specific lifestyle (time spent outside at home) and dietary (consumption of coffee, beans, and fruit juices) factors were identified that substantially increased the exposures of these participants to pyrethroids insecticides in residential settings. To better characterize the daily exposures of adults to pyrethroids (that breakdown to form 3-PBA), it is recommended that future studies use 24-h urine, drinking water, and food measurements as well as information regarding time spent outside and the consumption frequency of specific foods and beverages, particularly coffee, beans, breads, and fruit juices.

## Figures and Tables

**Table 1:** Urinary 3-PBA concentrations in 50 adult participants over a 24-h monitoring period ^a,b^.

3-PBA	*n* ^c^	% ^d^	Mean ± SD ^e^	GM ^f^	Minimum	Percentiles				Maximum
						25th	50th	75th	95th	
ng/mL	50	98	2.43 ± 2.59	1.68	<0.25	0.99	1.41	2.87	8.85	13.7
ng/mg	50	98	2.85 ± 3.04	1.74	<0.25	0.94	1.53	3.57	10.1	13.6

^a^ A subset of the urine data was used from Morgan et al. [20]. These data represent 24-h urine samples collected from adults during sampling days 2–3 of week 1. Completion rates (88%) were the highest for this first sampling interval compared to the other sampling intervals (six total). ^b^ Urine data values were averaged per person (range = 3–11) over the 24-h period. ^c^ Number of 24-h composited urine samples. ^d^ Percentage of 24-h composited urine samples ≥ limit of quantification (LOQ) of 0.25 ng/mL. ^e^ Arithmetric mean and standard deviation. ^f^ Geometric mean.

**Table 2:** 24-h urinary 3-PBA levels (ng/mL) in Ex-R adults by selected demographic and lifestyle factors.

Factors	*n* ^a^	% ^b^	3-PBA	
			GM (95% CI) ^c^	*p*-Value
**Demographic factors**				
Sex				
Female	30	60	1.73 (1.18–2.53)	0.769
Male	20	40	1.60 (1.28–2.01)	
Age				
≤30 years old	27	54	1.59 (1.27–1.99)	0.64
>30 years old	23	46	1.78 (1.22–2.61)	
Race ^d^				
White	25	69	1.86 (1.49–2.33)	0.699
Black	11	31	1.64 (1.12–2.40)	
BMI ^e^				
Underweight/Normal (<25.0)	17	34	1.78 (1.11–2.84)	0.734
Overweight (25.0–29.9)	13	26	1.85 (1.26–2.72)	
Obese (≥30.0)	20	40	1.49 (0.97–2.30)	
**Lifestyle factors**				
Sampling season ^f^				
Spring	16	32	1.53 (0.96–2.43)	0.803
Summer/fall	15	30	1.63 (1.04–2.57)	
Winter	19	38	1.85 (1.21–2.82)	
Insecticide use at home ^g^				
Yes	28	56	1.41 (1.12–2.40)	0.102
No	22	44	2.09 (1.67–2.62)	
Time spent outside at home				
<0.5 h	34	68	1.32 (1.05–1.65)	0.002
≥0.5 h	16	32	2.80 (1.91–4.10)	

^a^ Number of adults. ^b^ Percentage of adults. ^c^ Geometric mean and 95th confidence interval. ^d^ Three other race categories (Hispanic, Asian, and Native American) were excluded from the analysis due to a small sample size (<7) by group. ^e^ Body Mass Index (BMI) categories. ^f^ Combined summer and fall seasons due to small sample sizes of nine and six participants, respectively. ^g^ Insecticide use by the homeowner within one month of field sampling.

**Table 3:** Urinary 3-PBA levels (ng/mL) in Ex-R adults by selected food item over the 24-h sampling period.

Dietary Factors	*n* ^a^	% ^b^	3-PBA	
			GM (95% CI) ^c^	*p*-Value
**Fruits**				
Apples				
0 times	35	70	1.56 (1.25–1.96)	0.383
≥1 time	15	30	1.97 (1.34–2.89)	
Bananas				
0 times	35	70	1.46 (1.16–1.83)	0.074
≥1 time	15	30	2.32 (1.58–3.40)	
Berries ^d^				
0 times	30	60	1.83 (1.46–2.29)	0.385
≥1 time	20	40	1.47 (1.01–2.16)	
Strawberries				
0 times	36	72	1.69 (1.35–2.11)	0.94
≥1 time	14	28	1.65 (1.13–2.42)	
**Vegetables**				
Beans ^e^				
0 times	34	68	1.49 (1.19–1.86)	0.141
≥1 time	16	32	2.17 (1.48–3.18)	
Carrots				
0 times	32	64	1.75 (1.40–2.19)	0.626
≥1 time	18	36	1.55 (1.06–2.27)	
Leafy ^f^				
0 times	23	46	1.82 (1.46–2.28)	0.518
≥1 time	27	54	1.56 (1.06–2.29)	
Onions				
0 times	32	64	1.56 (1.25–1.96)	0.439
≥1 times	18	36	1.90 (1.30–2.78)	
Potatoes ^g^				
0 times	34	68	1.72 (1.38–2.16)	0.745
≥1 times	16	32	1.58 (1.08–2.32)	
Peppers ^h^				
0 times	33	66	1.84 (1.47–2.30)	0.286
≥1 times	17	34	1.40 (0.96–2.05)	
Tomatoes				
0 times	20	40	1.62 (1.30–2.03)	0.831
≥1 times	30	60	1.71 (1.17–2.51)	
**Meats**				
Baked or grilled chicken/turkey				
0 times	37	74	1.87 (1.49–2.34)	0.127
≥1 times	13	26	1.23 (0.84–1.80)	
Fried chicken or chicken nuggets				
0 times	40	80	1.60 (1.28–2.01)	0.458
≥1 times	10	20	2.01 (1.37–2.94)	
Ham or lunch meats				
0 times	35	70	1.66 (1.33–2.08)	0.923
≥1 times	15	30	1.71 (1.16–2.50)	
**Dairy**				
Cheeses				
0 times	33	66	1.53 (1.22–1.91)	0.285
≥1 time	17	34	2.01 (1.37–2.94)	
Yogurts				
0 times	32	64	1.65 (1.32–2.07)	0.876
≥1 time	18	36	1.72 (1.17–2.52)	
**Grains**				
Breads ^i^				
0 times	27	54	2.01 (1.61–2.52)	0.1
≥1 time	23	46	1.35 (0.92–1.98)	
Cereals				
0 times	36	72	1.66 (1.33–2.08)	0.896
>1 time	14	28	1.72 (1.17–2.52)	
Spaghetti and other pastas				
0 times	33	66	1.63 (1.30–2.04)	0.754
≥1 time	17	34	1.77 (1.21–2.59)	
**Other Foods**				
Salty snacks ^j^				
0 times	24	48	1.69 (1.35–2.11)	0.956
≥1 time	26	52	1.67 (1.14–2.44)	
Sweet snacks ^k^				
0 times	27	54	1.53 (1.23–1.92)	0.425
≥1 time	23	46	1.86 (1.27–2.73)	
Nuts and nut butters				
0 times	34	68	1.54 (1.23–1.93)	0.308
≥1 time	16	32	2.01 (1.37–2.94)	
**Beverages**				
Soft drinks (sugar)				
0 times	35	70	1.50 (1.14–1.98)	0.169
≥1 time	15	30	2.16 (1.40–3.33)	
Soft drinks (sugarless)				
0 times	38	76	1.76 (1.35–2.29)	0.486
≥1 time	12	24	1.44 (0.87–2.38)	
Coffee				
0 times	27	54	1.42 (1.00–2.02)	0.137
≥1 time	23	46	2.03 (1.52–2.72)	
Fruit juices				
0 times	37	74	1.50 (1.19–1.90)	0.122
≥1 time	13	26	2.29 (1.27–4.14)	
Milk				
0 times	31	62	1.87 (1.45–2.41)	0.247
≥1 time	19	38	1.40 (0.89–2.21)	
Water ^l^				
≤1 time	13	26	1.66 (0.99–2.79)	0.963
>1 time	37	74	1.68 (1.29–2.19)	

^a^ Number of adults. ^b^ Percentage of adults. ^c^ Geometric mean and 95th confidence interval. ^d^ Includes blackberries, blueberries, cherries, cranberries, and raspberries. ^e^ Includes baked, black-eyed, garbanzo, green, lima, and soybeans. ^f^ Includes spinach, lettuce, collard greens, and turnip greens. ^g^ Includes French fries, tater tots, hash browns, mashed, baked, and salad. ^h^ Includes red, green, and yellow varieties. ^i^ Includes white/wheat breads, rolls, English muffins, biscuits, bagels, and scones. ^j^ Includes chips, crackers, pretzels, and rice cakes. ^k^ Includes cakes, doughnuts, cookies, pastries, granola bars, and cereal bars. ^l^ Includes city, well, vitamin and seltzer water.

**Table 4:** Final regression model of factors impacting ln urinary 3-PBA levels in 50 Ex-R adults over a 24-h sampling period ^a^.

Factors	Factor Type	β Coefficient	Standard Error	*p*-Value
Intercept	---	−1.90	0.922	**0.046 ^b^**
24-h time spent outside ^c^	Lifestyle			**0.0006**
≥0.5 h		0.778	0.211	
<0.5 h		0 (ref.)		
Beans	Dietary			0.100
Yes		0.404	0.240	
No		0 (ref.)		
Breads	Dietary			**0.019**
Yes		−0.490	0.200	
No		0 (ref.)		
Coffee	Dietary			**0.007**
Yes		0.661	0.232	
No		0 (ref.)		
Fruit juices	Dietary			0.066
Yes		0.442	0.234	
No		0 (ref.)		
Creatinine level (mg/dL) ^d^	---	0.403	0.187	**0.037**

^a^ The *R*^2^ = 0.44. ^b^ Statistically significant variables are in bold text. ^c^ At home, only. ^d^ Continuous variable (log-transformed).
